# The Order-Disorder Continuum: Linking Predictions of Protein Structure and Disorder through Molecular Simulation

**DOI:** 10.1038/s41598-020-58868-w

**Published:** 2020-02-07

**Authors:** Claire C. Hsu, Markus J. Buehler, Anna Tarakanova

**Affiliations:** 1 Department of Electrical Engineering and Computer Science, Massachusetts Institute of Technology, Cambridge, MA USA; 2 Laboratory for Atomistic and Molecular Mechanics, Department of Civil and Environmental Engineering, Massachusetts Institute of Technology, Cambridge, MA USA; 3 Department of Mechanical Engineering, University of Connecticut, Storrs, CT USA; 4 Department of Biomedical Engineering, University of Connecticut, Storrs, CT USA

**Keywords:** Biochemistry, Biophysics, Molecular biology, Structural biology, Engineering

## Abstract

Intrinsically disordered proteins (IDPs) and intrinsically disordered regions within proteins (IDRs) serve an increasingly expansive list of biological functions, including regulation of transcription and translation, protein phosphorylation, cellular signal transduction, as well as mechanical roles. The strong link between protein function and disorder motivates a deeper fundamental characterization of IDPs and IDRs for discovering new functions and relevant mechanisms. We review recent advances in experimental techniques that have improved identification of disordered regions in proteins. Yet, experimentally curated disorder information still does not currently scale to the level of experimentally determined structural information in folded protein databases, and disorder predictors rely on several different binary definitions of disorder. To link secondary structure prediction algorithms developed for folded proteins and protein disorder predictors, we conduct molecular dynamics simulations on representative proteins from the Protein Data Bank, comparing secondary structure and disorder predictions with simulation results. We find that structure predictor performance from neural networks can be leveraged for the identification of highly dynamic regions within molecules, linked to disorder. Low accuracy structure predictions suggest a lack of static structure for regions that disorder predictors fail to identify. While disorder databases continue to expand, secondary structure predictors and molecular simulations can improve disorder predictor performance, which aids discovery of novel functions of IDPs and IDRs. These observations provide a platform for the development of new, integrated structural databases and fusion of prediction tools toward protein disorder characterization in health and disease.

## Introduction

Intrinsically disordered proteins (IDPs) make up 35 to 45% of proteins contained within eukaryotes, and sequences with an IDR (intrinsically disordered region) longer than 30 residues occur twice as frequently in eukaryotic proteins than in sets of randomly selected proteins^1,2^. Disordered regions fulfill a variety of functions: short linear motifs play a role in targeting for post-translational modifications or cell signaling^3–5^ and longer regions promote molecular recognition and protein-protein interactions^6,7^, among others. IDPs and IDRs can serve as flexible linkers between structured regions or as flexible binding sites for ligands^6^. Some IDPs undergo a disorder-order transition upon binding to other proteins through molecular recognition features (MoRFs), amphipathic regions within longer disordered regions^6,8^. The nature of the disordered regions is key to the resulting function of the protein: the length of the disordered region, the amount of disorder, and the specific location of the disordered regions all influence the functional role of the protein^6^.

**Figure 1:**
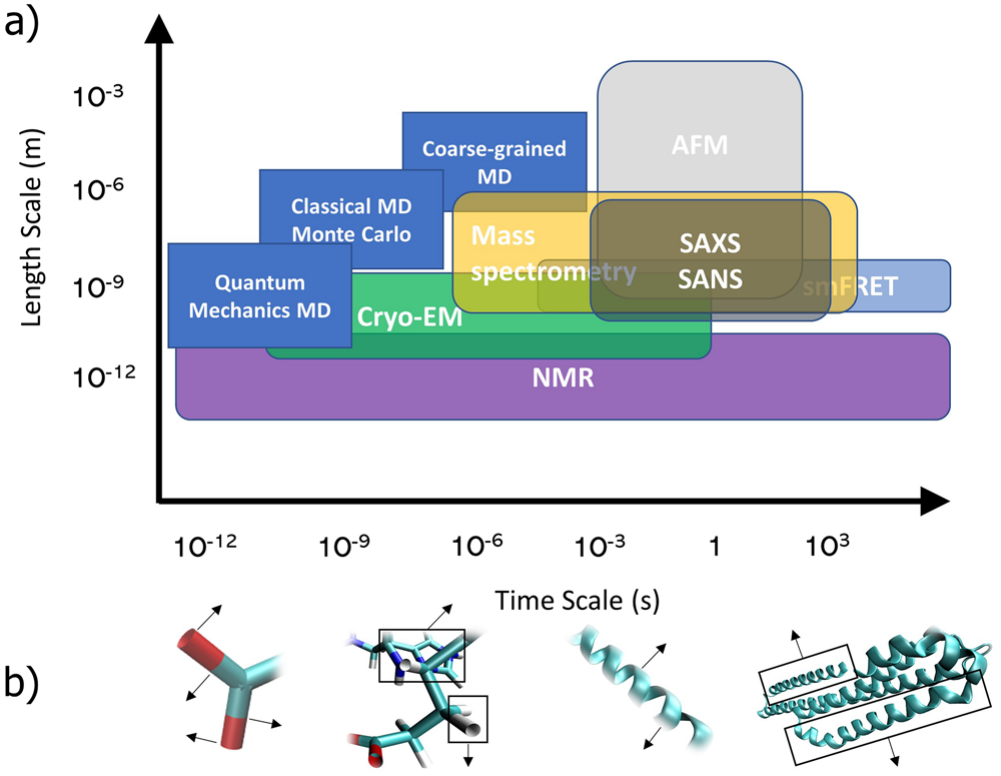
(**a**) Experimental and simulation techniques used to define protein structure and dynamics at different time and length-scales. (“MD” – molecular dynamics; “AFM” – atomic force microscopy; “SAXS” – small angle X-ray scattering; “SANS” – small angle neutron scattering; “EM” – electron microscopy; “NMR” – nuclear magnetic resonance spectroscopy; “smFRET” – single molecule fluorescence resonance energy transfer) (**b**) Movement at different length-scales (bonds, side chains, residues, and domains) that can be characterized. Visualization with VMD^103^.

Biological implications of IDPs and IDRs range from cell signaling to cell cycle control^6,9^. IDPs and IDRs play a role in numerous diseases, examples including the tau protein in Alzheimer’s^10^, aggregate proteins in Parkinson’s disease^11^, and several driver proteins and prion-like regions in neurodegenerative diseases such as amyotrophic lateral sclerosis (ALS)^12–14^. Recent studies have also connected structural disorder to drug design applications^15^, as characterization of the dynamics of a disease-associated IDP may guide ligand selection during drug development, and have identified the role of disorder in enzymic function^16^. The strong link between protein function and protein disorder motivates a deeper and more fundamental characterization of IDPs and IDRs for discovering new functions and relevant mechanisms. However, the structure of IDPs and IDRs and associated functions remain hard to detect - these proteins tend to evolve faster than structured proteins at the sequence level, so there is less functional information to derive from homologues^6,17–20^. In addition, many current experimental techniques fail to accurately characterize IDPs and IDRs due to their dynamic nature^21,22^. Some techniques also have resolution or timescale constraints (as illustrated in Fig. 1), which can affect the ability to capture disorder on residue length scales or longer timescales^23,24^.

### Characterizing protein structure and disorder

A variety of experimental techniques (as detailed in Fig. 1) are used to characterize the structure of proteins, with variable applicability to rigid and flexible proteins: some methods can capture conformational transitions of IDPs and IDRs while others fail to describe dynamics at all. Some methods used to characterize protein structure include X-ray crystallography, NMR spectroscopy, mass spectrometry (MS) techniques, electron microscopy, and small-angle X-ray scattering (SAXS).

X-ray crystallography is one of the most commonly used techniques for structural characterization of proteins found in the PDB (Protein Data Bank)^25^, suitable for proteins that can be successfully bound to ordered crystals. However, X-ray crystallography generally fails to determine the structure of dynamic regions^22^, which leads to regions of missing electron density in resolved protein structures.

Recent advances in NMR spectroscopy have contributed to the characterization of protein ensembles with increasing resolution^26–28^. NMR spectroscopy can successfully capture the dynamics of protein structures^28^, and integrative models now combine different techniques with NMR to more accurately characterize dynamic features of a protein^29–33^. Recent advances have proposed kinetic protein crystallography, combining high resolution static imagery from X-ray crystallography with lower resolution 3-D structural ensembles from NMR spectroscopy, to provide an improved description of protein structure than either method individually^29,30^. Other methods propose coupling NMR with molecular dynamics simulations to capture conformational heterogeneity of proteins^31–33^.

Mass spectrometry can be used to capture conformational intermediates^34^. For example, ion-mobility mass spectrometry (IM-MS) with electrospray ionization uses the resulting charge state distribution to determine conformations and disorder^34–36^. Hydrogen/deuterium-exchange mass spectrometry (HDX-MS) captures the dynamics of IDPs well^37–39^, as protein conformation affects rates of exchange, especially in cases when other methods fail to characterize highly disordered regions^22^. HDX-MS is notably useful in describing regions of protein-protein interaction, where IDPs may undergo binding^40^.

Recent advances in single-particle electron cryo-microscopy (CryoEM), where multiple protein conformations can be isolated, have generated images of disordered proteins with up to 4 Å resolution^41–43^. Small-angle X-ray scattering (SAXS) and small-angle neutron scattering (SANS) can characterize flexible IDPs and IDRs and determine protein compactness^44,45^, and combined with other high-resolution techniques like NMR, small-angle scattering techniques can derive structural information on multiple length-scales^24^.

Single molecule fluorescence techniques such as single molecule Förster resonance (smFRET) have helped to describe protein ensembles by capturing long-range transitions between IDP and IDR configurations^23,46^. While newer methods may contribute accurate characterizations of multiple IDP and IDR conformations, models may still conflict with one another, which has led to a growing number of studies adopting integrative methods, utilizing multiple techniques to generate models at multiple resolutions^47^.

### Cataloguing disorder

Functional proteins may exist in numerous conformations: the Protein Quartet model proposes solid and ordered, liquid-like and disordered, gas-like extended disordered, and pre-molten globule disordered states, which suggests different levels of disorder linked to the various IDP and IDR functions^48,49^. Yet many existing definitions of disorder used in training modern disorder predictors or classifying regions in disorder databases still utilize a polarized ordered or disordered designation^50–56^. Proteins generally lie on an order-disorder continuum^21^, a description which recognizes that there may be significant intermediate stages that can have functional implications. To capture this, structural ensembles of proteins are often characterized, capturing different folded or unfolded states of a protein and its dynamic motion, through a growing arsenal of NMR techniques^21^. However, large structure databases such as the PDB still lack an extensive number of such ensembles, and its experimental data often fails to report multiple protein conformations. NMR, while better suited for IDPs and IDRs, still sometimes fails to assign quickly fluctuating disordered regions^22^. Newer databases specific to storing protein ensembles, such as the Protein Ensemble Database (PED)^57^ and the Protein Order and Disorder Database (PODD)^21^, and databases specific to storing protein disorder annotations, such as DisProt^58^, are also much smaller in size than the PDB, on an order of 100 to 1,000 times – for instance, the PED contains around 24 protein ensembles and the PODD contains over 5000 two-dimensional protein ensembles. DisProt catalogs over 2,000 disordered regions. Thus, there exists a disconnect between the state of protein databases and the order-disorder continuum that can capture the full spectrum of protein structure and dynamics.

To better address the state of disorder prediction, below we evaluate the strengths and limitations of currently available structure prediction methodology. Secondary structure prediction has evolved with the growth of machine learning and predictive algorithms, whose varying performances may be leveraged to complement disorder predictions.

### A brief history of protein secondary structure prediction

The solution to predicting protein secondary structure has evolved quickly over the past few decades, making strides in optimizing input features and model architecture. Early predictors utilized neural networks^59–62^ and support vector machines^63^, with Rost and Sander^64^ and Zvelebil *et al*.^65^ as some of the first to use multiple sequence alignments as an input feature into their neural network. Jones then introduced PSI-BLAST output matrices, which contain sequence conservation information based on similar amino acid sequences, as a new input feature in PSI-PRED^66^, setting the precedent for nearly all future predictors. Cuff and Barton then combined Hidden Markov Model (HMM) profiles with the PSI-BLAST profile to create the Jnet prediction method, which uses two artificial neural networks^67^. Other recent work used machine learning to capture structural features of proteins and applied it to the design of new protein sequences^68^.

These early predictors formed the foundation for many modern predictors, as research has accelerated in recent years. Input had largely been considered on a residue-by-residue basis, until the idea of utilizing local and global contexts within protein sequence arose. Convolutional neural networks (CNNs) provide more information on surrounding sequence and structure by using a sliding window to capture a fuller local context^69^. Compared to unmodified position-specific scoring matrix (PSSM) profiles, CNN features as inputs have improved prediction accuracy by up to 4%^70^.

Recurrent neural networks (RNNs) have also improved prediction accuracy by taking global sequence context and nonlocal interactions into account. Other models attempt to capture more information around each residue by using sliding windows containing surrounding regions for each residue, but RNNs can retain information from any part of the previously seen sequence. To solve the disappearing or exploding gradient problem often found with RNNs, models have adopted gate and memory structures, such as GRUs^71^ and long short-term memory (LSTM) units^72,73^. Many models built to predict protein structure are also bidirectional recurrent neural networks (BRNNs), traversing the sequence in the forward and backward direction. Examples include SSPRO^74^, an ensemble of 100 BRNNs, and SPIDER3^73^, a combination of two LSTM BRNNs and two fully connected layers.

To address dependencies on adjacent secondary structure labels, many models also infer secondary structure from nearby secondary structures, in addition to local or global PSI-BLAST profile patterns. Baldi *et al*.^74^ introduced a template-based method, which uses secondary structure of homologous proteins as a template for prediction of other structures. Conditional neural fields^75^ can also take advantage of surrounding labels, or surrounding secondary structure, to influence prediction of other labels.

Since each method provides a unique piece of information (e.g. local context, global context, nearby structure dependencies), many models also combine different methods. For example, DeepCNF^76^ utilizes deep convolutional neural networks instead of shallow neural networks in its conditional neural field. Li and Yu combined multiscale CNNs with stacked BGRUs to learn both multiscale local contexts and nonlocal interactions^71^.

Most predictors use PSSM profiles as input features derived from a nonredundant subset of the PDB, such that the subset maintains some level of sequence dissimilarity in the form of percentage identity cutoff. Additional inputs include the raw amino acid sequence (in one-hot encoding), Hidden Markov Model (HMM) profiles, and other physio-chemical annotations or properties of the protein. Common benchmark protein sets include CASP^77^ sets or CB513^67^, which also remain under some sequence identity cutoff from the training set.

In 2001, Rost theorized a limit on prediction accuracy of 88%^78^, accounting for state-of-the-art predictor performance (PSIPRED, JPred2) at the time. Reasons for such a limit include limitations and ambiguities on structure determination through X-ray or NMR methods, limitations and hard-coded threshold values in assignment algorithms (such as in the DSSP^79^ algorithm), and, of particular relevance to this study, the dynamic nature of protein structure.

The above predictors utilize static structure information from the PDB, which creates a disconnect between structure predictors and protein disorder. Such a disconnect may still be leveraged to provide additional disorder information, especially in the case of varying disorder definitions and predictive techniques, as we will demonstrate.

### Predicting Protein Disorder

To address the challenge of protein disorder prediction, several disorder predictors have been created, all of which take advantage of different sources of data. Disorder has long been characterized as the absence of atomic coordinate information in native structures determined by X-ray crystallography due to the flexibility of the protein in that region, deeming it invisible in crystallographic electron density maps. As a result, many predictors of disorder in IDPs and IDRs focus on labeling regions of missing electron density as regions of disorder^50,51,80^. As discussed above, a number of techniques has been effective in detecting disorder to develop structural ensemble datasets, thereby capturing conformational variability and disorder in flexible proteins. More recently, annotations taken from disorder databases accumulate such experimental data. These varying definitions of disorder, however, affect the performance and transitivity of predictors. As a result, the current individual definitions of disorder may have to be expanded or combined to account for the diverse functionalities of IDPs and IDRs and the order-disorder continuum.

Disorder predictor development ranges from deterministic biophysical models to trained machine learning algorithms. Predictors such as IUPred^81^ utilize existing structural data for a rule-based system that predicts disorder in a novel protein given its sequence, usually by aiming to minimize energy or favoring specific amino acid pairs over others. IUPred specifically uses an energy estimation method, where pairwise potentials are determined between pairs of amino acids, and the model parameters are derived only from globular proteins from the PDB. As in the case of secondary structure predictors, machine learning methods have become increasingly popular, as seen with the development of predictors such as DISOPRED3 (neural network)^51^, DisEMBL (neural network)^82^, PONDR-VSL2B (SVM)^54^, and DeepCNF-D (conditional neural field)^50^. Combining different types of methods results in meta-predictors, such as PONDR-FIT^83^ or GSmetaDisorder3D^84^, which take advantage of multiple predictors specialized in identifying different types of disordered regions (e.g. length) or trained using different methods (e.g. energy functions, machine learning).

In addition to inconsistent definitions of disorder, the difficulty of building a disorder predictor also lies in the intrinsic relationship between disordered region length and protein function^85^. Specifically, short IDR identification remains an integral part of disorder prediction, as these regions can act as motifs or serve as linkers. However, there has often been a disparity between short disordered region prediction (<10 AA) and long region prediction using general disorder predictors. Due to biases in training data or feature selection^54^, predictors trained on longer regions of disorder tended to perform more poorly while predicting short disordered regions^86^, so to compensate, some predictors train separately on datasets of different disordered region length. For instance, the PONDR predictor family contains separate predictors for long ( > 30AA) and short regions (VSL2-L and VSL2-S, respectively). Both achieve an overall accuracy of over 80% for their respective length regions^54^. However, for general length-independent predictors, shorter regions tend to yield higher prediction accuracy than longer regions^55,87^.

### Databases of protein disorder

Recent interest in IDPs and IDRs as important functional proteins has sparked the development of disordered protein databases, which often combine multiple experimental techniques. As mentioned previously, DisProt, the PED, and the PODD all catalog a number of disordered regions or protein ensembles, leveraging experimental techniques such as small-angle X-ray scattering, NMR, and SAXS.

With the increase in disorder predictor performance, some databases now combine predicted disorder annotations with any available experimental information. For instance, MobiDB 3.0 accumulates information from DisProt, UniProtKB and FuzDB for general disorder annotation, as well as disorder predictions from IUPred, VSL2b, DisEMBL, and other disorder predictors in its three-layered annotation scheme^53^. MobiDB contains disorder information from predictions for over 80 million proteins. D^2^P^2,^ ^52^ similarly utilizes multiple predictors, including IUPred, VSL2b, and Espritz predictors, and combines them into a disorder agreement metric. It contains prediction information on over 10 million proteins. A comprehensive review of disorder predictors and databases can be found in He (2009)^9^ and Meng (2017)^1^.

As these databases were more recently developed (D^2^P^2^ first developed in 2012, MobiDB in 2012 and DisProt in 2006) compared to protein databases for traditional folded protein structure, the number of proteins with experimental, manually curated information in these databases (rather than predicted data) is not yet on the scale of databases such as the PDB or UniProt, which contain over 100,000 structures. This disparity leads to a lack of catalogued disorder information which could provide insight into novel protein functions.

### Leveraging structural data for disorder predictions

Currently, static structural data has limited use for disorder prediction because disorder inherently relies on the dynamic nature of proteins. Here we explore novel approaches to leverage static structural data and structure prediction algorithms for extracting additional disorder information that existing disorder predictors fail to capture, through molecular simulation and structure prediction error.

Disordered regions within folded proteins can be used to train disorder databases. The structure predictor itself can give us information about disorder within the static structure. Because structure predictors are often trained on evolutionary data to recognize structure from homologous proteins, disordered regions will inherently perform worse because disordered regions are known to evolve at a faster pace. In addition, structure predictors are also generally trained on static structural information, which fails to capture dynamics and flexibility of a protein, especially in disordered regions.

A recent study evaluated 26 different disorder predictors, which demonstrated large variability in disorder predictions^56^. This variability could be attributed to the different inherent definitions of disorder, different training datasets, or different predictor specialties, as detailed earlier. More concerning, however, is the discovery of the under-prediction of disorder in many disorder predictors – for instance, DISOPRED3, a predictor used in this study, tended to bias towards ordered labels. This disparity calls for additional sources of data that can be leveraged for disorder information in the case where disorder predictors fail.

Recent work has studied the link between structure and disorder^21,28^, through the development of the s2D method, for example, which concurrently predicts disorder and structure using NMR spectroscopy. In this work, we look at the link between secondary structure and disorder predictor results, exploring these results in comparison to dynamic protein fluctuation signatures determined from molecular simulation of sample proteins. We highlight regions of high flexibility revealed by molecular simulations that disorder predictors fail to capture. We find, consistently across all types of prediction methods, that areas of poor structure predictor performance may suggest high flexibility or disorder.

## Methods

Experiments were conducted using CullPDB^88^, a set of 11,154 proteins sharing no more than 25% sequence identity. This dataset was split into training and test sets by isolating 15% (1673 proteins) of the original set as the test set. The CullPDB derived training set was further filtered to remove any sequences sharing more than 40% identity with any protein in the test sets. 8-class labels to represent protein secondary structure were generated using DSSP^79^, with missing DSSP labels assigned as coiled residues.

To look at samples with a varied structure content, a non-redundant test set of 1673 proteins was split into 5 bins of increasing helix (including 3_10_, α, π helices) and beta (beta strand and bridges) content. The helix/beta content was quantified by counting chains of consecutive amino acids of either helical or beta secondary structure based on DSSP, exponentially increasing with chain length. Normalization of the score was performed by dividing total content score by overall sequence length. Chain lengths were kept within 200 ± 20 residues.

We considered three disorder predictors with different input data and training methods: IUPred (long)^81^, DISOPred^51^ and DISOclust^89^. IUPred uses physical properties of amino acid pairs in the sequence to determine order/disorder. DISOPred3 uses a consensus method between DISOPred2 and an additional SVM classifier for protein binding. DISOclust utilizes variability in predictions from ModFOLD2clust, which compares 3D models of a protein, to judge disorder.

Four secondary structure predictors are also considered: DeepCNF^76^, SPIDER3^73^, SSPRO8^74^, and 2D-CNN^70^. DeepCNF and the 2D-CNN are both machine learning-based models, using convolutional neural nets trained on PSSM matrices, while SPIDER3 uses a bidirectional recurrent neural net with LSTM cells trained on PSSM matrices, in addition to the HMM profile and physio-chemical properties of the amino acid sequence. SSPRO8 also utilizes a bidirectional recurrent neural network but takes in structural similarity as an additional parameter. These models were trained on culled versions of the PDB, employing a cutoff sequence identity to eliminate redundancy in training sets. To evaluate sampled proteins, the web servers for the first three predictors were used, and 2D-CNN was implemented locally as described in Supplemental Information online. A summary of the secondary structure and disorder predictors used in the study is included in Supplementary Table S1.

Molecular models of five representative protein structures with PDB IDs 3PLW^90^, 2R6V^91^, 1DZF^92^, 3HZ8^93^, 3UMH^94^ were considered. It should be noted that despite the use of MD for identifying regions of disorder, there are weaknesses associated with the method, including incomplete sampling and dependence on starting structure. Residues missing from the PDB file were excluded from the simulation and results. All simulations were carried out using GROMACS version 5.1.2^95^. Each structure was placed into a rectangular water box with periodic boundary conditions. The CHARMM27 force field was used, which includes CHARMM22 and CMAP for proteins^96^. While CHARMM36m could have been used to provide improved correlation of the data, the less computationally expensive CHARMM22/CMAP force field was sufficient to demonstrate a proof of concept, as we are able to capture fluctuations and correlated (short) disordered regions. Each molecule was fully solvated using the TIP3P water model^97^ and neutralized by adding the appropriate number of chloride counter ions. Frames were saved every 2 ps for analysis. Each structure was first minimized through the steepest descent algorithm to ensure no steric clashes. Then, each structure was simulated for 1 μs in an NVT ensemble at 310 K. The time step used was 2 fs. The Berendsen thermostat^98^ was used for temperature coupling. The LINCS^99^ algorithm was used to constrain covalent bonds with hydrogen atoms. The short-range electrostatic interactions and Lennard-Jones interactions were evaluated with a cutoff of 10 Å. Particle-mesh Ewald summation^100^ was used to calculate long-range electrostatic interactions with a grid spacing of 1.6 Å and a fourth order interpolation.

A total of 1500 frames was extracted out of each 1 μs simulation. The last 300 ns of each simulation was sampled to extract a molecular longevity metric. For each residue in each sample, structural longevity was measured as the average duration during which the secondary structure DSSP assignment remained constant, with a score of 1 equivalent to a constant structure through the 300 ns, and a score nearing 0 indicating structural fluctuation within every 200 ps range. Intermediate values were determined by considering stability of a DSSP assignment at a given residue, increasing exponentially with longer durations of consistent DSSP label. The longer the assignment remained consistent, the higher the 0–1 value assigned.

1 $${\rm{Longevity}}\,{\rm{at}}\,{\rm{residue}}=\frac{avg(number\,frames\,with\,consistent\,DSSP\,label)}{total\,frames}$$

We used in-house TCL and Matlab scripts to perform all analysis. All simulations were completed using the Extreme Science and Engineering Discovery Environment (XSEDE).

## Results

**Figure 2:**
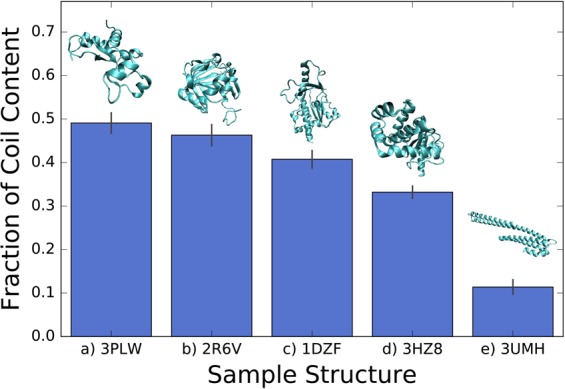
% coil content vs order counter for sampled molecules, 3PLW^90^ (**b**) 2R6V^91^ (**c**) 1DZF^92^ (**d**) 3HZ8^93^ (**e**) 3UMH^94^. The five proteins are ordered from greatest to least coil content.

**Figure 3:**
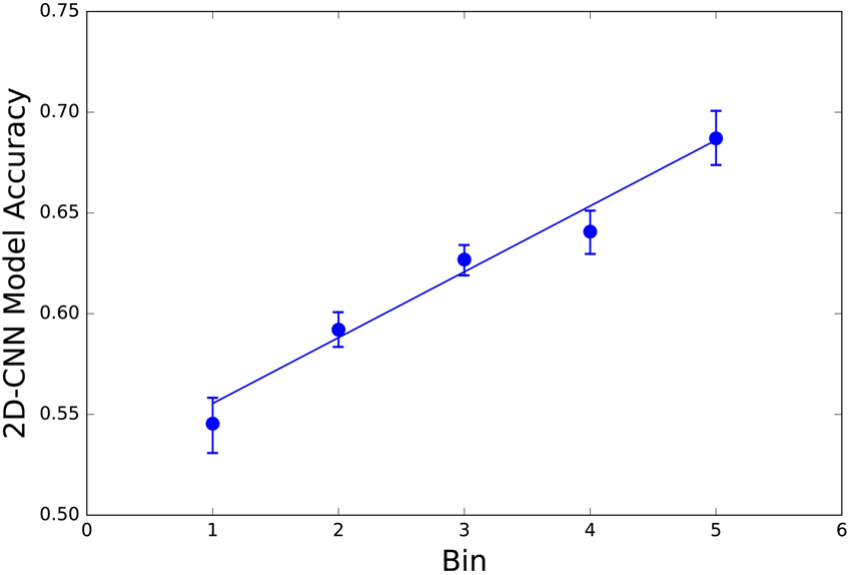
Prediction accuracy for all proteins in each of five bins (the test set split into bins of differing helix/beta content) in increasing order, using 2D-CNN^70^ as implemented in Supplemental Information. Confidence interval = 0.95.

**Figure 4:**
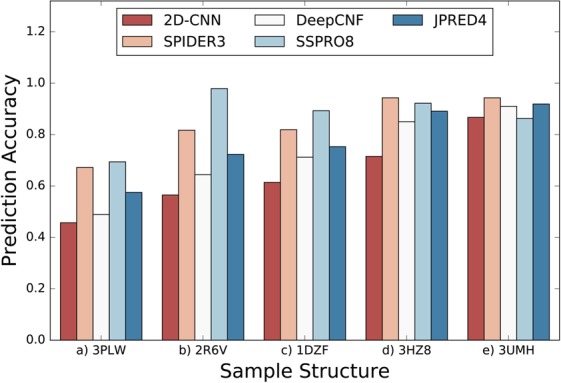
Prediction Accuracy of 5 samples (**a**) 3PLW^90^ (**b**) 2R6V^91^ (**c**) 1DZF^92^ (**d**) 3HZ8^93^ (**e**) 3UMH^94^), with increasing order (sampled proteins).

**Figure 5:**
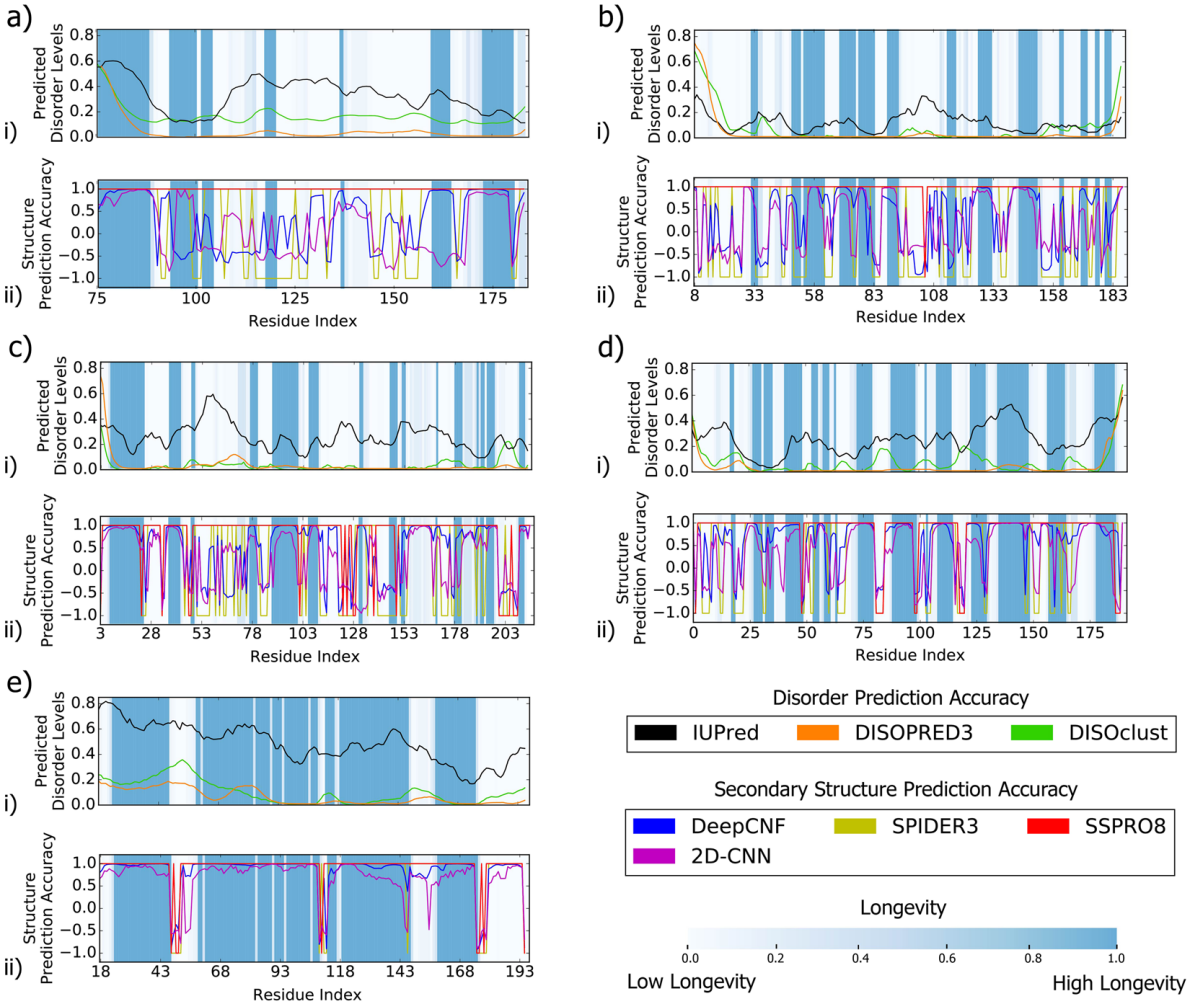
Predicted disorder with IUPred^81^, DisoPRED^51^, and DISOclust^89^ predictors (i) and secondary structure prediction accuracy based on SPIDER3^73^, DeepCNF^76^, 2D-CNN^70^, and SSPRO8^74^ predictors (ii) highlighted with molecular structure longevity through molecular dynamics simulation (i, ii) for (**a**) 3PLW^90^ (**b**) 2R6V^91^ (**c**) 1DZF^92^ (**d**) 3HZ8^93^ (**e**) 3UMH^94^. For longevity, blue regions indicate higher longevity regions while white regions indicate lower longevity regions.

**Figure 6:**
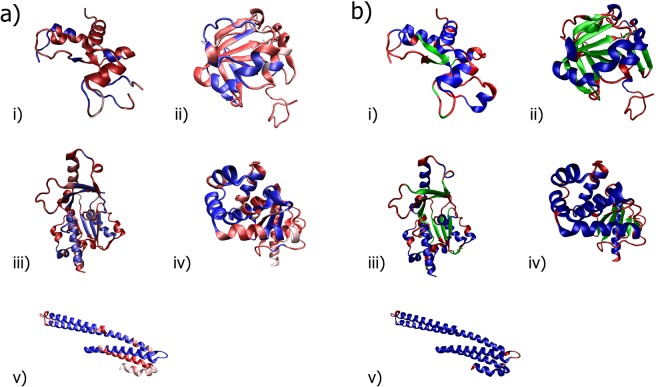
Models of the five sampled proteins in this study: (i) 3PLW^90^ (ii) 2R6V^91^ (iii) 1DZF^92^ (iv) 3HZ8^93^ (v) 3UMH^94^. (**a**) Molecular longevity on a red/blue scale for low/high structural longevity. (**b**) DSSP assignments with red (coiled), green (beta), and blue (helix) structures.

**Figure 7:**
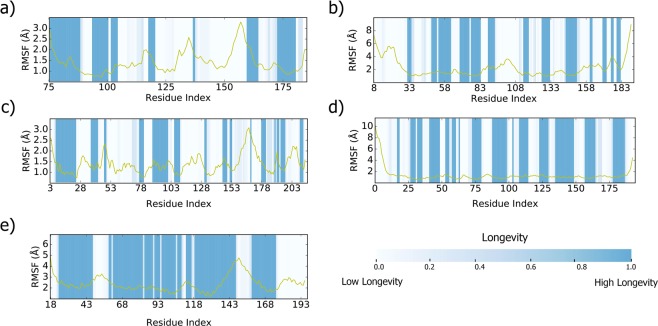
Root mean square fluctuation (RMSF) per-residue plots highlighted with molecular structure longevity through molecular dynamics simulation for (**a**) 3PLW^90^ (**b**) 2R6V^91^ (**c**) 1DZF^92^ (**d**) 3HZ8^93^ (**e**) 3UMH^94^. For longevity, blue regions indicate higher longevity regions while white regions indicate lower longevity regions.

In this study, we first split protein samples – a non-redundant test set of 1673 proteins – content into bins of increasing disorder, where ordered structure is defined as helix (including 3_10_, α, π helices) and beta structures, and evaluate different predictive model performance on each bin. We then display the predictor performance alongside disorder predictions and molecular longevity data, identifying key regions of correlation and disagreement. Figure 2 evaluates content of sampled representative proteins from each test set, Figures 3 and 4 study structure predictor performance, and Figures 5–7 closely examine the structure and disorder of each sampled protein across its sequence. We first define the level of disorder in a protein by its secondary structure content. When dividing the test set into bins, we separate protein sets based on a decreasing coil content and corresponding increasing helix/beta content. 1672 proteins in the test set were divided into five bins of size 214, 452, 508, 233, and 265 proteins, in increasing helix/beta content. In general, increased coil content tends to align with increased disorder and associated flexibility in a protein^50,101^, so our test set separation corresponds to an increasing degree of disorder. Figure 2 captures the increasing helix and beta content and corresponding decreasing coil content across the five sampled proteins, one from each test bin. Bin level is assigned according to secondary structure content – bin 1 is represented by structure 3PLW, bin 2 by structure 2R6V, bin 3 by structure 1DZF, bin 4 by 3HZ8, and bin 5 by 3UMH.

Structure prediction model performance was then evaluated for all proteins in each of the five bins. Cumulatively, the convolutional neural net model proposed by Li *et al*.^71^ predicts secondary structure for proteins in the last bin of protein samples (highest order) with 15% higher accuracy than proteins in the first bin (least order) (bin 5 compared to bin 1 in Fig. 3). Prediction accuracy is directly correlated to the degree of disorder in the molecule.

Across another four published methods with varying machine learning techniques, more accurate predictions are found for increasingly ordered proteins (Fig. 4). Note that Fig. 4 depicts prediction accuracy for sampled proteins from each bin while Fig. 3 shows average model (2D-CNN) accuracy for all proteins in each bin. We find that accuracy increases from the most disordered to least disordered proteins in both cases. JPred4 and SPIDER3 both utilize Q3 (3-state secondary structure, with states coil, beta, and helix) DSSP labels, which could account for higher performance than the more specified Q8 (8-state secondary structure, with states 3_10_ helix, α helix, π helix, beta bridge, extended strand, turn, bend, and loop) labels used by 2D-CNN and DeepCNF. We note that SSPRO8 deviates from a linear trend as the model takes sequence-based structural similarity into account. However, all five predictors generally increase from a prediction accuracy of 60% to 80% from the most disordered to least disordered sampled proteins. We also note a direct correlation between prediction accuracy in Fig. 4 and the sample helix and beta content in Fig. 2.

We compare per-residue results from four secondary structure prediction algorithms: SPIDER3^73^, DeepCNF^76^, 2D-CNN^70^, and SSPRO8^74^ for five representative protein structures with PDB IDs a) 3PLW^90^ b) 2R6V^91^ c) 1DZF^92^ d) 3HZ8^93^ e) 3UMH^94^. The top panel in Fig. 5(a–e) displays the per-residue predictions from three disorder predictors: IUPred (long), DisoPRED, and DISOclust for comparison. The bottom panel in Fig. 5(a–e) corresponds to the results of the four secondary structure predictors. The blue highlight in both panels in Fig. 5(a–e) displays the longevity of secondary structure based on molecular dynamics simulation results. Longevity is defined as average number of frames of consistent DSSP label divided by total number of frames, as in Eq. (1).

Accuracy of secondary structure prediction and degree of disorder is found to be consistently inversely correlated. The three disorder predictors considered show some correlation among themselves, but disagree in key regions (Fig. 5(a–e)(i)). DISOclust peaks tend to be more pronounced compared to more subtle peaks in IUPred or DISOPRED. For example, in Fig. 5(a)(i), the DISOclust peaks mirror those of DIOSPRED at residues 110–120 and 155–165 but have larger amplitudes, and are much more defined than the less clear peaks in IUPred predictions. As noted in Nielsen 2019^56^, DISOPRED tends to under-predict disorder, which may explain its comparatively lower overall disorder predictions. At protein regions of high disorder, sample proteins demonstrate dips in secondary structure prediction accuracy and confidence based on all four structure predictors considered here (Fig. 5(a–e)(ii)). Model accuracy is represented by positive (correct) and negative (incorrect) values. Model per-residue confidence is determined using maximum class probability derived from n-class output as values and is represented on a 0 to +/−1 scale (farther from 0 suggesting higher confidence).

In regions of increased predicted disorder, especially around peaks predicted by DISOclust and DISOPRED3 (whose predictions have generally lower values among the three disorder predictors considered), SPIDER3 tends to predict less accurately **(**Fig. 5(a–e)(i-ii)). These peaks in disorder are likely significant, as those regions often also display significant peaks in IUPred, which suggests consensus. For instance, in Fig. 5(a)(i), peaks in disorder from residue 110 to 120 translate to a band of low secondary structure prediction accuracy (Fig. 5(a)(ii)). Similar trends are found in Fig. 5(b)(i-ii) at residue 90 to 100 and Fig. 5(c)(i-ii) at residue 45 to 70. These regions are also consensus regions of disorder for three disorder predictors considered (Fig. 5(a-e)(i)). We also observe that molecular longevity (visualized in Fig. 6(a–b)(i–v)) tends to correlate well with structure predictor performance across all five sampled proteins. However, molecular longevity results highlight regions not well-identified by disorder predictors but better correlated with structure predictor accuracy. For instance, Fig. 5(c)(i-ii) at residue 110 to 128 displays a band of poor SPIDER3 performance and low longevity, but no corresponding peak in disorder.

DeepCNF predictions display similar correlations to disorder predictions as do the SPIDER3 predictions **(**Fig. 5(a–e)(i-ii). Figure 5(b)(i-ii) at residue 133 to 145 displays weak DeepCNF prediction confidence (light red and blue colors suggest high uncertainty in the predicted DSSP label at these regions) and low longevity. Figure 5(c)(i-ii), from residue 110 to 140, also displays low structure predictor accuracy and longevity, but no significant peak in predicted disorder, other than a minor peak from IUPred. However, at consensus regions of peak disorder (as before), we find weak DeepCNF performance (e.g. Figure 5(b)(i-ii) res. 90–100, Fig. 5(c)(i-ii) res. 45–70, Fig. 5(e)(i-ii) res. 45–55). Interestingly, longevity and DeepCNF predictor accuracy tend to align with DISOclust predictions, even when there is no consensus disorder prediction at these regions.

2D-CNN prediction accuracy also aligns well with molecular longevity results, and peaks in disorder match with poor 2D-CNN performance and low longevity regions (Fig. 5(a–e)(i-ii)). We again observe key regions of poor 2D-CNN confidence or inaccuracy and low molecular longevity that do not have a corresponding disorder consensus (e.g. Figure 5(c)(i-ii) res. 103–150, Fig. 5(a)(i-ii) res. 125–150). In addition, while 2D-CNN performance again aligns well with DISOclust and most DISOPRED results, it has mixed alignment with IUPred predictions. For instance, at some peaks, there is good correlation between IUPred and poor predictor performance (Fig. 5(b)(i-ii) res. 58–70), but at other regions, an IUPred peak with no consensus from the other two disorder predictors does not translate to poor structure predictor performance (Fig. 5(d)(i-ii) res. 130–145). Notably, some regions that dip suddenly in IUPred predicted disorder experience small periods of poor 2D-CNN predictor performance and short molecular longevity at the edges of such regions (e.g. Figure 5(a)(i-ii) res. 90–110, Fig. 5(b)(i-ii) res. 70–85).

SSPRO8 predictions contain far less error than the other three predictors, but around major consensus peaks such as the ones mentioned before, there are regions of poor SSPRO8 predictor performance (Fig. 5(a–e)(i-ii)). However, some key regions already highlighted do not demonstrate any dips in SSPRO8 predictor performance when the other three predictors did demonstrate dips (e.g. Figure 5(a)(i-ii) res. 110–120, Fig. 5(c)(i-ii) res. 53–70). As for the previous three structure predictors, SSPRO8 performance also dipped during bands of short molecular longevity.

Comparing prediction accuracy or confidence to per-residue structure longevity (Fig. 5(a–e)(ii) in molecular dynamics simulation shows that poorly predicted regions align with regions that display shorter average structural longevity (Fig. 6(a–b)(i–v)), which indicates more motion and flexibility in the region during molecular simulation. All predictors demonstrate this trend, either with predictors predicting incorrectly or with low confidence at these regions of highly dynamic motion. Combined with the correlation between disorder and structure predictions, we find many consensus peaks in disorder align with dynamic regions as determined by molecular simulation across all sampled protein models.

However, some key regions display a high degree of flexibility without a corresponding peak in disorder predictors. Areas with average structural longevity reaching zero suggest consistent fluctuation in the protein structure in simulation and should suggest a high degree of disorder. Despite this, some regions display low SS predictor performance and structural longevity, but no significant signal in any disorder predictor (e.g. Figure 5(c)(i-ii) res. 100–150). Other regions display varying SS predictor performance and low structural longevity with conflicting signals in disorder predictors (Fig. 5(a)(i-ii) res. 90–100).

Regions of higher longevity per-residue generally correspond to helix and beta structures while regions of lower longevity align with coiled regions, as labeled by DSSP (Fig. 6(b)(i–v)). We also observe that in increasing structural order, overall structure longevity also increases (Fig. 6(a–b)(i–v)).

Additionally, we confirm our longevity measure with RMSF per-residue plots (Fig. 7). Peaks and higher-value regions in RMSF generally align with low longevity regions, such as in Fig. 7(a), res. 135–150, Fig. 7(c), res. 50–70, and Fig. 7(e), res. 105–115.

## Discussion

Labeled disordered regions within the structures considered, as determined by disorder predictors, all align with regions along the sequence that undergo more structural fluctuation, quantified as low molecular longevity, and poor secondary structure predictor performance. Yet, some regions with similar trends in longevity and secondary structure predictor performance do not have a corresponding significant peak in disorder. This disparity suggests that current disorder predictors may fail to capture some disordered regions with high molecular motion, especially in the case of globular proteins like the ones tested in this study. Furthermore, several regions did not reach a consensus on disorder levels among disorder predictors, which aligns well with variability results and under-prediction of disorder found in Nielsen 2019^56^. However, as secondary structure predictors tend to predict incorrectly or predict with low confidence at these key regions, secondary structure predictor performance may be used as an additional marker of disorder that current disorder predictors fail to capture.

In our analysis, we find that trends in longevity and SS predictor performance align most closely with the disorder prediction from DISOclust. The DISOclust method is based off variation in residue positions in multiple fold recognition models, given the assumption that on a per-residue basis, residues that are more structurally aligned are more ordered. This characterization compares most closely with our molecular longevity definition, where high longevity corresponds to conserved secondary structure on a per-residue basis. Since the three disorder predictors root from three inherently different definitions of disorder, occasionally there is not a consensus on whether a region is predicted to be disordered.

We find consensus among secondary structure predictors: while the individual algorithms or machine learning method used are different, much of the input data and input format are similar, which results in similar outputs across predictors. Most structure predictors utilize a subset of the PDB or UniProt databases to train models, and they almost always use a PSSM format to better represent an “input sequence” for prediction. However, disorder predictors do not form a consensus in many regions considered, due to the number of different algorithm formats (deterministic vs learned) and input data sources (fold recognition data vs DisProt and PDB data). As a result, structure predictors can help identify and clarify disordered regions in which different predictors may not reach a consensus.

These trends exist for structures with varying structural content, as proteins were sampled from bins separated by helix/beta/coil content. Upon inspection of the protein chains (Fig. 6(a–b)(i–v)), many regions of low molecular longevity are coiled regions connecting more structured regions, possibly serving as flexible linkers. Compared to results shown in (Fig. 5(a–e)(i-ii)), these regions are also potentially disordered, suggesting a partial order-disorder continuum within even well-characterized globular proteins. Such regions may include residues 48 to 50 in 3UMH and residues 119 to 121 in 3HZ8, which connect helix regions (Fig. 6(b)(iv-v)) and display weak structural predictor performance and disorder peaks (Fig. 5(d,e)(i-ii)). For instance, molecular dynamics simulation can provide insight into short disordered regions within globular proteins that are difficult to identify but may have biological implications. In addition, molecular dynamics may help to extrapolate incorrectly assigned structures within disordered regions in globular proteins and clarify the propensity of structure or lack thereof within these regions. Extending this approach to existing IDPs and IDRs, this method can identify disordered regions not previously highlighted through other predictors.

Our longevity measure taken from molecular dynamics simulations matches closely with RMSF per-residue plots also derived from simulations (Fig. 7), which further supports the usage of molecular dynamics as an additional determinant of disordered regions in proteins. Both approaches characterize protein flexibility, notably in regions where existing predictors miss disordered regions, as mentioned previously. RMSF per-residue values can also help detail different types of high longevity regions. For instance, high longevity ordered regions would experience low fluctuation, but high longevity disordered regions (e.g. some coiled regions) would experience higher fluctuation. In the specific cases in this study, most coiled regions experienced low longevity – because our longevity measure accounted for Q8 labels, many of these regions alternated between coil and turn state, which are generally both considered coiled in other contexts.

Because secondary structure predictors are still largely based on static structural databases, incorrectly predicted regions may suggest higher degrees of flexibility and disorder. While current databases grow to include dynamic ensemble information, static structural information can still be leveraged to make conclusions about disorder, especially at disordered regions that current disorder predictors fail to identify. With this insight, future studies may consider utilizing such data to form more complete disorder predictions or database entries. As current databases take advantage of multiple sources for disorder prediction, the addition of molecular dynamics information would contribute to a more thorough analysis of a structure’s disorder. As experimental techniques for disorder classification grow, using molecular dynamics and static structure predictor performance as an indicator of disorder can contribute to disorder determination to characterize the role of IDPs and IDRs in disease, cell signaling, and drug design.

## Conclusion

We provide an overview of current experimental methods for the determination of IDPs and IDRs as well as the current state and shortcomings of disorder prediction. To contribute to the more accurate identification of disorder, we have presented secondary structure predictions and molecular longevity measurements as additional markers of disorder, especially in cases where existing disorder predictors fail to reach a consensus. Regions that are marked disordered by multiple predictors also experience poor secondary structure predictor performance and low per-residue structural longevity, but some regions that are marked disordered by only one or two predictors can be further clarified through molecular longevity data. This method can contribute to identification of disordered regions in proteins where disorder may be more subtle or under-predicted, as shown in the five sampled globular proteins, which can contribute to the identification of additional disordered regions key to biological functions.

## Supplementary information

Suplemental Information.

## Data Availability

Structures used for molecular simulations are available in the Protein Data Bank, online at https://www.rcsb.org/. All predictors are available online as described in the corresponding references. The culled datasets used to conduct experiments are available at http://dunbrack.fccc.edu/Guoli/pisces_download.php.
