# Supplementary material for: The Order-Disorder Continuum: Linking Predictions of Protein Structure and Disorder through Molecular Simulation
Source: Sci Rep. 2020 Feb 7;10:2068. doi: 10.1038/s41598-020-58868-w (PMC7005769; doi:10.1038/s41598-020-58868-w)
Supplement: Supplementary file 1 — Suplemental Information. [file 41598_2020_58868_MOESM1_ESM.docx]

**Supplemental Information**

Claire C. Hsu, Markus J. Buehler, Anna Tarakanova. The Order-Disorder Continuum: Linking Predictions of Protein Structure and Disorder through Structural Dynamics Determined by Molecular Simulation.

**CNN Implementation**

The convolutional neural network from Liu et al. (2D-CNN ^1^) was implemented to evaluate predictor performance locally on full test set bins, as modelled in **Figure 3**, rather than sampled representative protein structures from each test bin. For each sequence, PSIBLAST ^2^ was used to generate a position-specific scoring matrix (PSSM) of size 20xN (N = length of sequence) with E-value threshold 0.001 and 3 iterations to search the PDB. The 20xN matrix was split into matrices of window size 21, such that the matrix was cut into a series of 20 x 21 matrices, with one centered on each residue. To account for edge cases, the initial 20xN was padded with columns of 0s on either end.

The convolutional neural network architecture utilizes two convolutional layers with sigmoid activation. The first layer has 96 filters of size 5x5 and the second layer has 24 filters of size 2x2. Between the layers, a max pooling layer with pool size 2x2 and stride 2 was used. Following the two convolutional layers, a fully connected layer with 512 units and a softmax layer formed the output. The softmax layer generates the probabilities for each of the 8 classes, with the SS prediction for a specific residue being the class with the highest probability.

The model trained on 3 iterations of the training set. In addition, a 50% dropout layer was used for the fully connected layer, and the Adam optimizer was used. A negative log likelihood loss function was used in the back-propagation step of model optimization.

| Method | Predictor Type | Description |
| --- | --- | --- |
| SPIDER3 | Structure | Long short-term memory bidirectional recurrent neural network trained iteratively to predict 3-state secondary structure. |
| DeepCNF | Structure | Deep convolutional neural field (a combination of conditional random fields and deep convolutional neural networks) trained to predict 8-state secondary structure. |
| 2D-CNN | Structure | Two-dimensional convolutional neural network trained to predict 8-state secondary structure. |
| SSPRO8 | Structure | Multi-stage predictor that combines bidirectional recursive neural networks trained on sequence similarity data and structural similarity data from the PDB to generate 8-state secondary structure predictions. |
| IUPred (long) | Disorder | Energy estimation-based predictor that utilizes differences in energy between ordered and disordered regions to quickly predict disorder. |
| DISOPRED3 | Disorder | Three neural network models (SVM model from DISOPRED2, neural network for long disordered regions, and nearest neighbor model) combined by a small neural network to form 0 to 1 disorder predictions. |
| DISOclust3 | Disorder | Unsupervised predictor of disorder based on per-residue variability in multiple fold recognition models (ModFOLDclust2). |

**Supplementary Table S1: Secondary structure and disorder prediction methods used in study, classified by method name and predictor type.**

**References**

1 Liu, Y., Chen, Y. & Cheng, J. in *2016 9th International Congress on Image and Signal Processing, BioMedical Engineering and Informatics (CISP-BMEI).* 1771-1775.

2 Altschul, S. F. *et al.* Gapped BLAST and PSI-BLAST: a new generation of protein database search programs. *Nucleic Acids Res* **25**, 3389-3402, doi:10.1093/nar/25.17.3389 (1997).
